# Towards the Automatic Detection of Pre-Existing Termite Mounds through UAS and Hyperspectral Imagery

**DOI:** 10.3390/s17102196

**Published:** 2017-09-24

**Authors:** Juan Sandino, Adam Wooler, Felipe Gonzalez

**Affiliations:** Robotics and autonomous systems, Queensland University of Technology (QUT), 2 George Street, Brisbane City QLD 4000, Australia; adam_wooler@yahoo.com.au (A.W.); felipe.gonzalez@qut.edu.au (F.G.)

**Keywords:** pre-existing termite mounds, UAV, hyperspectral camera, machine learning, image segmentation, support vector machines

## Abstract

The increased technological developments in Unmanned Aerial Vehicles (UAVs) combined with artificial intelligence and Machine Learning (ML) approaches have opened the possibility of remote sensing of extensive areas of arid lands. In this paper, a novel approach towards the detection of termite mounds with the use of a UAV, hyperspectral imagery, ML and digital image processing is intended. A new pipeline process is proposed to detect termite mounds automatically and to reduce, consequently, detection times. For the classification stage, several ML classification algorithms’ outcomes were studied, selecting support vector machines as the best approach for their role in image classification of pre-existing termite mounds. Various test conditions were applied to the proposed algorithm, obtaining an overall accuracy of 68%. Images with satisfactory mound detection proved that the method is “resolution-dependent”. These mounds were detected regardless of their rotation and position in the aerial image. However, image distortion reduced the number of detected mounds due to the inclusion of a shape analysis method in the object detection phase, and image resolution is still determinant to obtain accurate results. Hyperspectral imagery demonstrated better capabilities to classify a huge set of materials than implementing traditional segmentation methods on RGB images only.

## 1. Introduction

**Figure 1:**
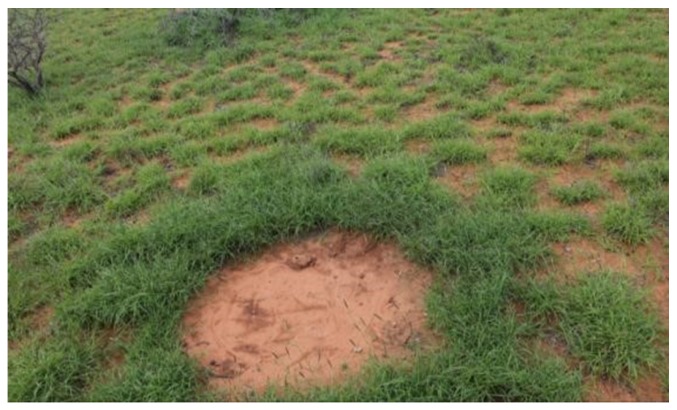
Pre-existing termite mound at Cape Range National Park, WA.

The contribution of termites to Australia’s environment is significant. Not only their colonies have positively contributed to enhancing the Australian landscape in savannah regions, but also they have been essential in decomposition processes [1,2,3]. Their diet allows the rapid disintegration of dead wood and plant debris, incrementing, therefore, nutrients’ quantification at the surroundings of their mounds [4,5]. Termites also play a significant role in creating dense nutrient patches through the erosion of soil from the aboveground mounds to the ground surrounding the termite mounds, as shown in Figure 1 [6,7]. Their nests provide the necessary alteration of the structure of many trees for the habitats of various animals including birds, reptiles and some mammals. They have also been vital in Aboriginal communities, whereby termites have been used for medicines, dietary supplements and mounds for campfire [8].

Termite distribution across Australia varies according to the average weather temperature in each one of its states. As an illustration, there is a very high termite density distribution on coastal areas of the mainland; high density towards the centre regions including Coastal New South Wales and Coastal South Australia; moderate in both deserts and cold regions such as Alice Springs and Canberra respectively; low and very low density in very cold regions including Melbourne and Tasmania [9,10].

Regardless of the positive or negative influence of termites in Australia, an adequate detection system would provide an accurate and efficient outcome for their study, monitoring and control. Amongst the various methods to accomplish this task, remote sensing is useful due to the easy management of large spatial distributions. For example, the contributions of Bunting et al. [11] demonstrated that through the use of image texture, vast improvements could be made in the classification of plant species to derive texture-based information. He et al. [12] reported what hyperspectral remote sensing could offer for invasion ecologists and review recent progress made in plant invasion research using hyperspectral remote sensing. Wallis et al. [13] demonstrated how remote sensing improved the performance for modelling biodiversity across space and time and for developing effective ecological indicators. As stated by Colomina and Molina [14], the use of UAS in remote sensing has led to a broad development of low-cost diverse agriculture applications, with plenty of facilities to build the products according to the needs of the offered services. Moreover, computer vision and open source philosophy has improved the manageability of UAVs and data processing. Therefore, the increased literature and technological developments in Unmanned Aircraft Systems (UAS) design and path planning, combined with hyperspectral imagery and machine learning approaches have opened the possibility of improving image quality [15] and increasing research outcomes in biosecurity [16], air quality, precision agriculture and environmental sensing [17,18,19,20].

Over the last few decades, there has been little historical research on the detection of mounds through remote sensing. What is remarkable, however, the work presented by Vogt [21] showing an approach of photointerpretation from satellite imagery with overall accuracy rates of nearly 50%, and similarly, the one by Vogt and Wallet [22] analysing the feasibility of using shape-based and object-based models with satellite imagery and how unique models must be collected at new studied areas to ensure accurate results. Therefore, in this paper, a first and novel approach towards the detection of pre-existing termite mounds with the use of a UAS, hyperspectral imagery, machine learning and digital image processing is intended. A new pipeline process is proposed to detect termite mounds automatically and reduce, consequently, detection times in a studied zone.

## 2. Methods and Materials

**Figure 2:**
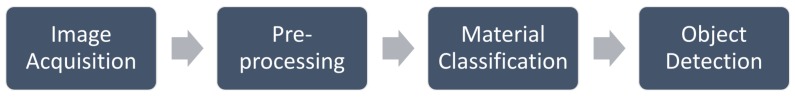
Primary proposed detection pipeline.

The primary method is contained in a pipeline composed of four main stages, as depicted in Figure 2. Firstly, there is an image acquisition phase, in which raw hyperspectral images are obtained by using a UAS. Later, a pre-processing stage is run to get a filtered hyper-cube, spatially cropped in regions with pre-existing termite mounds. Afterwards, a classification process is executed to extract valuable layers amongst the cropped image. Finally, a segmentation stage is carried out to filter termite mounds from other objects with similar material characteristics yet different shapes. Hence, a new algorithm is proposed to perform the classification and segmentation processes automatically.

### 2.1. Site

**Figure 3:**
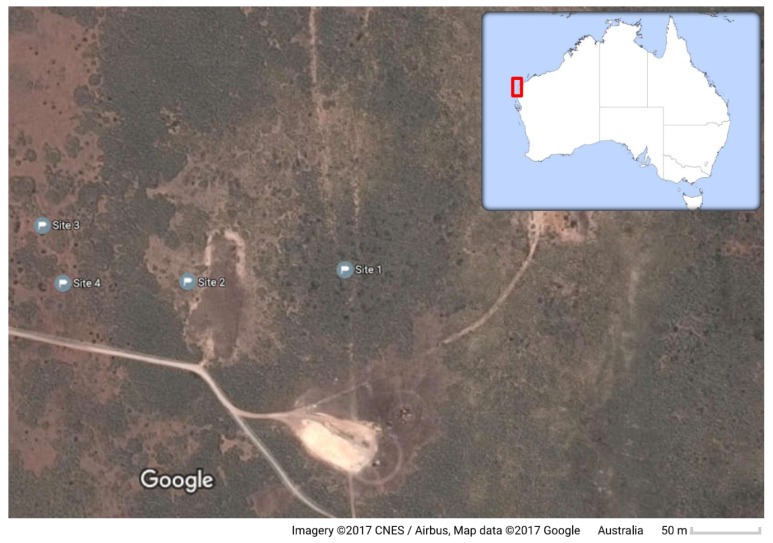
Primary location of the studied sites at Cape Range National Park, WA.

As shown in Figure 3, four sites with pre-existing termite mounds at Cape Range National Park, Western Australia, Australia (−22.190138, 113.865371), were chosen for this study. Each area comprises bush lands, buffel grass, eroded soil regions and remains of decomposed wood branches. The field trip was performed on 10 July of 2016, and a couple of mission routes were conducted between 1:00 p.m. and 2:00 p.m. Meteorological conditions included open sky, 46% average relative humidity, SE winds between 17 and 26 km/h, 0.0 mm rainfall and 21.2 °C maximum temperature [23].

### 2.2. Image Sensors

A Headwall Nano Hyperspec hyperspectral camera was utilised to generate information regarding the spectrum for each of the pixels in the studied region. This camera supports up to 274 spectral bands, wavelengths ranging from 385 nm–1000 nm (VNIR), 2.2 nm dispersion per pixel, 300 Hz maximum frame rate, 640 spatial bands and 480 GB of storage capacity. Additionally, a Canon EOS 5DS R digital camera was also used in order to capture high-resolution images from the same mission route and identify by photointerpretation the present pre-existing termite mounds in the studied area to save their GPS coordinates into a register file. The camera specifications include 50.6 MP resolution, 28 mm focal length, ISO-400 speed, full frame CMOS sensor of 36 mm by 24 mm, 625 μs exposure time and a GPS sensor.

### 2.3. UAV and Sample Acquisition

**Figure 4:**
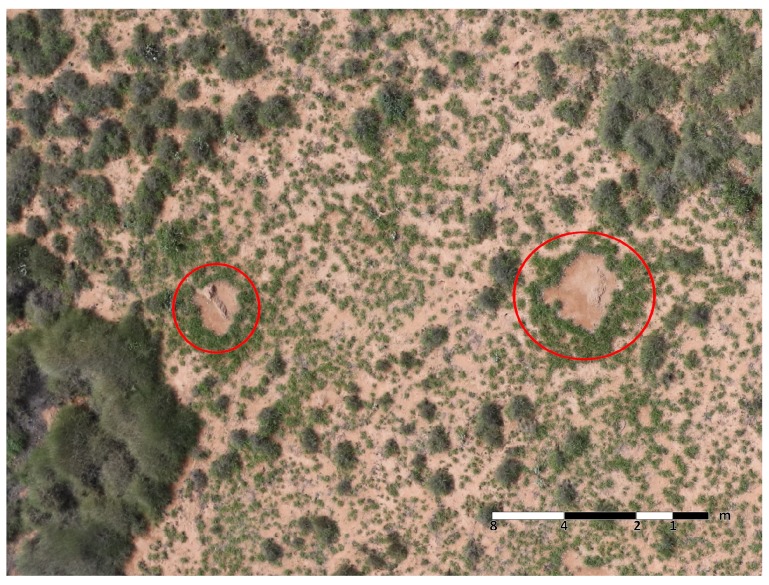
Highlighted pre-existing termite mounds in the planned mission route at −22.190228 latitude, 113.865197 longitude and 68.3 m altitude.

A Hexa-Rotor DJI S800 UAV, featuring a customised integrated gimbal system, high-performance brushless motors, dimensions of 1180 mm × 1000 mm × 500 mm and a total weight of 3.9 kg cameras included, flew on the mentioned site following a mission route in DJI Ground Station 4.0 software. The mission followed an altitude of 66.9 ± 4.6 m, overlap of 80%, side lap of 50% and 4.5 m/s mean speed for a path length of 6.6 km. During the sampling period, the vertical and horizontal Ground Sample Distances (GSD) of the RGB and hyperspectral images were approximately 1.0152 cm/pixel and 4.7 cm/pixel, respectively. By performing manual interpretation on the acquired samples from both cameras, a total of 23 pre-existing termite mounds were identified and targeted for this study. The mounds feature a mean rectangular area of 1.951 m^2^ and oval-based and diamond-based shapes in their centre, where vegetation density was virtually null and, conversely, a large grass concentration at their outer boundaries as illustrated in Figure 4.

### 2.4. Software

Raw hyperspectral data were firstly ortho-rectified. Afterwards, various regions of interest (ROI) were extracted by checking their coordinates with the recorded digital image dataset. Next, each ROI was processed with Scyven Software [24]. Based on the freeware Scyllarus™ toolbox provided by Data61|CSIRO, this application is capable of reading native HDR files and executing many hyperspectral image processing techniques. Amongst them, spatial and spectral filtering, reflectance recovery, material recovery and material classification through Support Vector Machines (SVM), Linear Spectral Unmixing (LSU) and Principal Component Analysis (PCA) methods were utilised. Scyven software allowed a complete analysis of various material reflectance spectra and identification of termite mound components. These spectra curves were accordingly applied at the classification stage as described in Section 2.5. The digital image processing segmentation methods were run with the implementation of OpenCV library [25]. A detailed description of the applied methods is described in the following section.

### 2.5. Algorithm

An algorithm was designed and tested to identify pre-existing termite mounds in given regions of interest (ROI). As shown in Algorithm 1, the process consists of a preprocessing, classification and a segmentation stage, mentioned beforehand in the primary pipeline method.
**Algorithm 1** Detection of pre-exiting termite mounds with hyperspectral imaging.
*Input:* raw hyperspectral image file
**Pre-processing**1.Calculate hyper-cube radiance2.Perform Orthorectification process3.Load GPS coordinates from register file, and obtain a ROI4.Apply closing operator once            ▷ structuring element: 3 × 3 rectangle
**Material Classification**5.Load illuminant spectrum from white reference6.Calculate reflectance7.Load material reflectance library8.Run SVM classifier9.Filter segmented material layers: “Eroded Soil” and “Light Grass”
**Object Detection**10.Apply Smooth-Median Filter on soil layer                 ▷ kernel size: 311.Apply closing operator on soil layer once      ▷ structuring element: 3 × 3 rectangle12.Create a temporal Image T113.T1← double dilation operator on soil layer      ▷ structuring element: 3 × 3 ellipse14.Apply Smooth-Median Filter on grass layer               ▷ kernel size: 315.Create a temporal Image T216.T2← AND operator between grass layer and T117.**if** Mean(T2) =0.0
**then return** null18.**end if**19.Find contours from the soil layer20.**for**
i←0,n
**do**                 ▷ n= number of detected soil contours21.  Select SC(i)                   ▷ SC(i)= soil contour at *i* index22.  Run MatchShapes method                   ▷ output: ratio23.  Psc← perimeter of SC(i)24.  **if**
ratio≤0.15
Psc≥10
**then**25.    Discard SC(i)26.  **else**27.    Find contours from the grass layer28.    **for**
j←0,m
**do**           ▷ m= number of detected grass contours29.      Select GC(j)             ▷ GC(j)= grass contour at *j* index30.     Pgc← perimeter of GC(j)31.     d← distance between GC(j) and SC(i) centroids32.     rad← radius of SC(i)33.     Prop←Pgc/Psc34.     **if**
d=1.2*rad (Pgc≥Psc || Prop0.8) **then**35.      ILD← Minimum intersection line distance between SC(i) and GC(j)36.      **if**
ILD=2.5
**then**37.        Draw contours at ROI image38.      **end if**39.     **end if**40.   **end for**41.  **end if**42.**end for**43.**return** ROI Image

#### 2.5.1. Pre-Processing

Based on the fact that raw imagery obtained by Headwall Nano Hyperspec camera contains intensity values in radiance per pixel in each of the 274 available wavelength bands and dimensional data of 640 by 7000 pixels, digital processing calculations were heavy due to significantly large data file sizes (≃3 GB). Several preprocessing methods were subsequently implemented to retrieve cropped orthorectified hyperspectral cubes, gathering smaller files that could be rapidly processed in Scyven and OpenCV.

**Figure 5:**
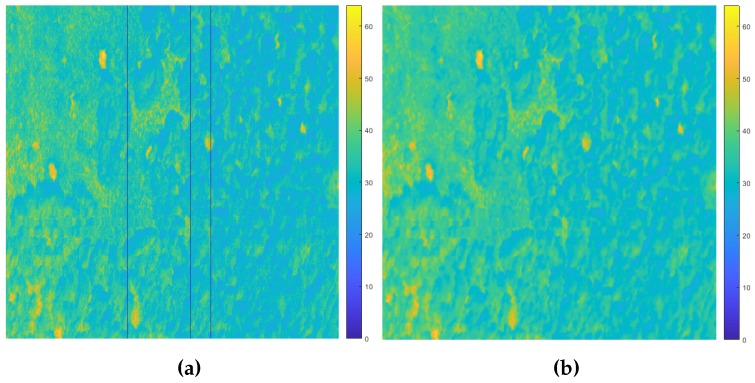
False colour representation of an ROI image in radiance during the pre-processing stage. (**a**) Raw image at 415 nm wavelength band. (**b**) Resultant filtered image.

With raw hyperspectral cubes as the starting point, radiance correction and orthorectification techniques were implemented by the inbuilt methods from Headwall Hyperspec^®^ III and SpectralView™ software. Then, the mentioned GPS coordinates database in Section 2.2 was read so as to crop and produce several ROIs with pre-existing termite mounds. After performing some random manual inspections, considerable noise and blur were discovered in many of the sample bands from each cropped hyper-cube. Consequently, a single iteration of the closing mathematical morphology operator was run firstly, using a rectangular 3 × 3 structuring element, as depicted in Figure 5.

#### 2.5.2. Material Classification

**Figure 6:**
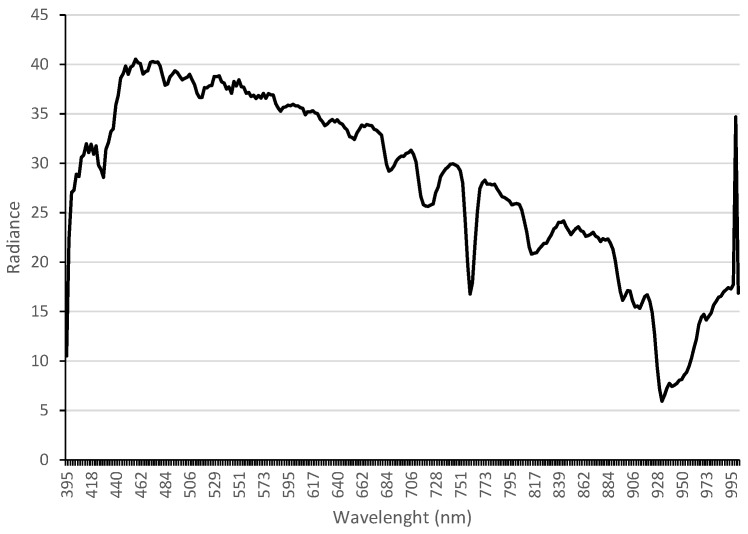
White reference spectrum acquired by the Headwall Nano Hyperspec camera.

In the first step of the classification process, the filtered hyperspectral cube in radiance was processed in order to obtain a new pixel-wise reflectance hyper-cube. Pixel reflectance was, therefore, obtained by importing the white reference spectrum into Scyven, acquired from the testing phase of the hyperspectral camera prior to the execution of the mission route, as shown in Figure 6. The use of a constant illuminant spectrum enhances the validation of the algorithm results instead of estimating dynamically various illuminant spectra [26] and, subsequently, different reflectance intensities for each loaded ROI into the software.

**Figure 7:**
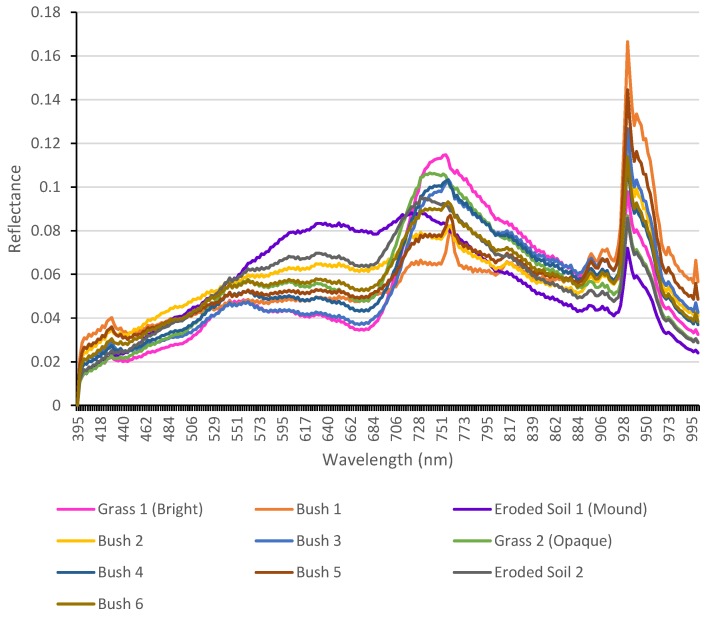
Clustered reflectance spectra of 10 materials from the demonstrative scene.

In order to run a machine learning classifier into the hyper-cube, it is required to load a set of material signatures that are wanted to be classified (targets). Prior and only once during this study, the spectra library was retrieved and clustered by selecting a demonstrative scene with an abundant amount of objects by using a deterministic annealing method [27]. Some of the input parameters for deterministic annealing are the maximum number of clusters, which was set to 10, a maximum temperature of 0.02, a minimum temperature of 0.00025, a cooling factor of 0.8, a split threshold of 0.8 and a maximum of 5 materials calculated per pixel. Figure 7 illustrates the spectral reflectance from the clustered materials.

**Figure 8:**
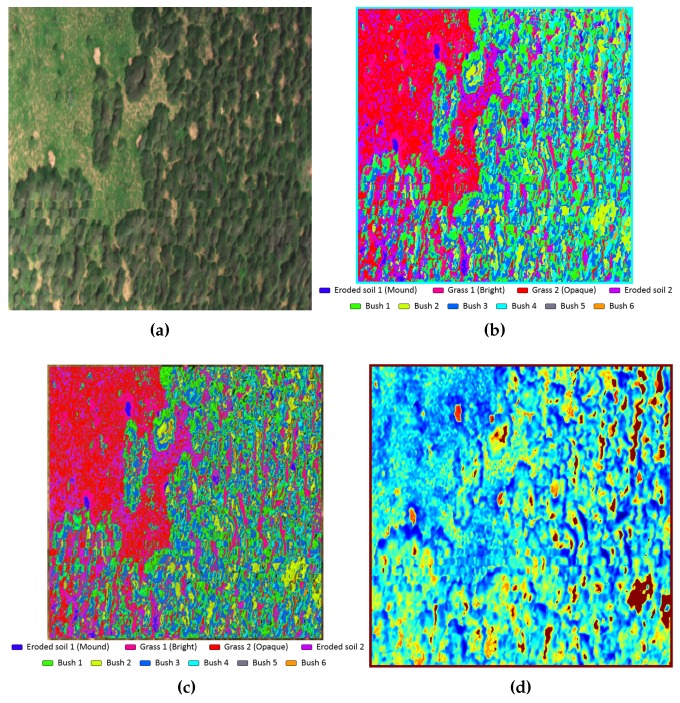
Classification algorithm outputs (**a**) Demonstrative ROI in RGB colour. (**b**) SVM output. (**c**) Linear Spectral Unmixing (LSU) output (**d**) PCA output in false colour.

Based on the materials library as mentioned above, Support Vector Machines (SVM), Linear Spectral Unmixing (LSU) and Principal Component Analysis (PCA) algorithms were tested prior the inclusion of only one of them in the proposed method. The spectra library mentioned above was loaded into the classifiers as targets to retrieve the outcomes that are shown, for example, in Figure 8. The SVM result (Figure 8b) depicts unique colour layers for each targeted material. In contrast, albeit similar, the LSU outcome (Figure 8c) presents certain dark areas, which represent pixels where none of the loaded materials had a significant correlation with the real signature at that location, resulting in colour layers with considerable noise. Finally, The first component of the PCA outcome illustrated in false colour (Figure 8d) is able to highlight alterations at the surroundings of each one of the mounds. Nevertheless, a segmentation process to identify mounds automatically based on PCA represents a challenging task due to the ambiguousness of the given results. In spite of the representation of high variations at the ROI, the available data were not conclusive in comparison to the classifiers as mentioned earlier, so that the primary test results were carried out by using the SVM classifier only.

**Figure 9:**
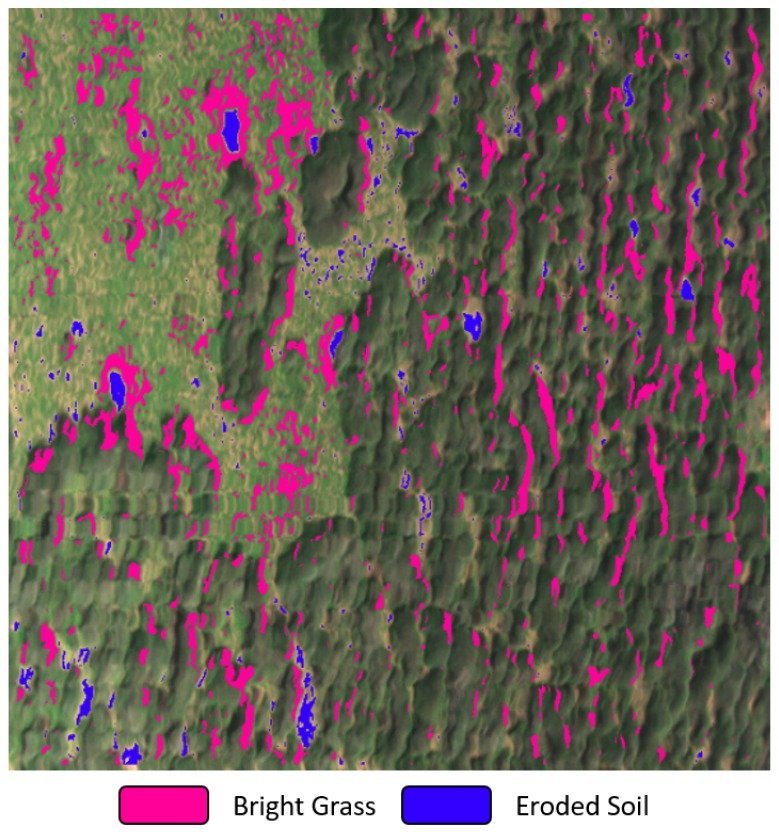
ROI with clustered termite mounds materials.

From the output image of the SVM classifier, two out of the ten layer materials were filtered in order to distinguish authentic termite mounds from other components that shared similar material properties. As illustrated in Figure 9, it is possible to separate by photointerpretation the areas that belong to termite mounds from others regarding bushes and other vegetation objects. Therefore, an additional image segmentation process is required to segment those areas with pre-existing termite mounds.

#### 2.5.3. Object Detection

**Figure 10:**
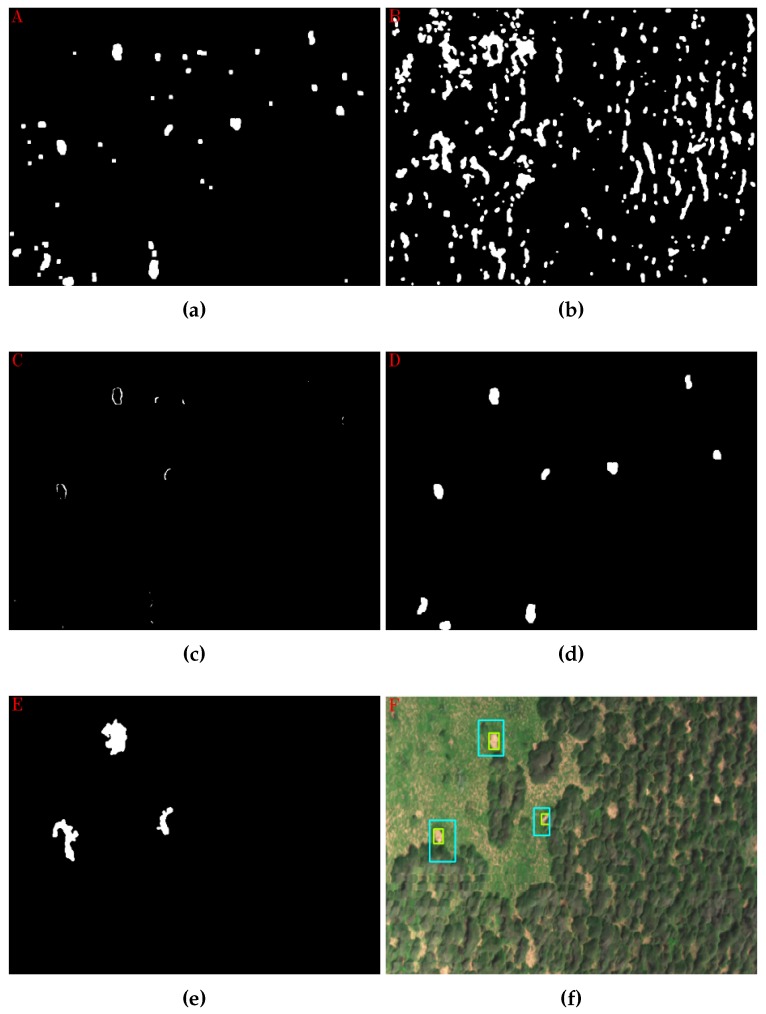
Main stages of the segmentation process. (**a**) Binary soil layer. (**b**) Binary grass layer. (**c**) AND logic operator between (**a**) and (**b**). (**d**) Filtered soil contours. (**e**) Filtered grass contours. (**f**) Identified contours in the ROI.

Steps 10–43 from Algorithm 1 permit the detection of pre-defined termite mounds based on a contour and shape analysis from the generated colour layers on previous steps. This phase allows one to count and display the location of the pre-existing termite mounds at each ROI, as depicted in Figure 10.

**Figure 11:**
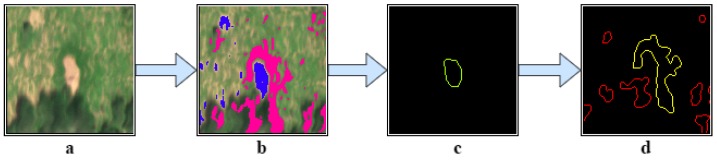
Contour analysis. (**a**) Cropped region. (**b**) classified material layers. (**c**) Detected contour at the soil layer. (**d**) Detected contours at the grass layer. Yellow contour: closest region from the contour (**c**).

The starting point is the manipulation of the binary colour layers from Figure 9. Filtration and morphological techniques were applied in order to attenuate their noise, as shown from Steps 10–14 (Algorithm 1) and depicted in Figure 10a,b. Following this, it was found that termite mounds could be identified from other objects by calculating the distance among their two layers. That gap is considerably small compared with other objects at the ROI. Figure 11 depicts, for instance, this finding using a cropped region with a mound at its centre.

Given a small frame of a termite mound at Figure 11a, the SVM classifier will generate a blue colour layer for the eroded soil signature and the pink one for the bright grass area. After detecting the contour from the blue layer at Figure 11c, all the mean distances between that contour and the exposed pink regions are calculated. If a distance threshold is satisfied (yellow contour from Figure 11d), the detected contour in Figure 11c will be identified as a pre-existing termite mound.

Accordingly, the pink colour layer was set to be slightly dilated in order to determine a good mound centre (Step 14). A temporary image is, therefore, computed by performing a logic AND operation between both layers with the aim to check whether the ROI indeed includes any region with mounds by calculating, thus, its mean intensity defined in Equation (1).

(1) $$Mean=\frac{\sum_{I=0}^{n}Src\left(I\right)}{n}$$

where Src belongs to the pixel value from the input image at the position *I* and *n* to the total number of pixels. If the mean threshold parameter from Step 17 of Algorithm 1 is not satisfied, the studied image will be discarded with zero found termite mounds as the final result. Figure 10c illustrates, for instance, the relevance of this operation.

**Figure 12:**
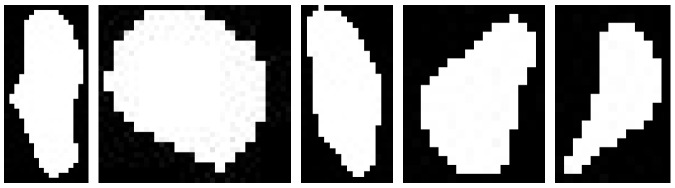
Termite mound templates for the shape matching method.

Once the first threshold condition is satisfied, a contour detection and shape analysis were run on the soil layer. Firstly, a contour detection algorithm from Suzuki and Abe [28] was executed at Step 19. Then, the detected soil contours were filtered using their shape properties by comparing their seven Hu moments [29] against some predefined pattern shapes. These patterns consist of a selected library of binary image templates that depicts key mound shapes, as illustrated in Figure 12.

By using a shape matching method in OpenCV, various contours with similar shapes from the loaded patterns can be detected regardless of their rotation, translation and scale properties. This method, mentioned in Step 22, returns a ratio value, calculated by following Equations (2)–(4).

(2) $$m_{i}^{S}=sign\left(h_{i}^{S}\right)\times log\left(h_{i}^{S}\right)$$

(3) $$m_{i}^{T}=sign\left(h_{i}^{T}\right)\times log\left(h_{i}^{T}\right)$$

(4) $$ratio=\sum_{i=1}^{7}\left|\frac{1}{m_{i}^{S}}-\frac{1}{m_{i}^{T}}\right|$$

where $h_{i}^{S}$ and $h_{i}^{T}$ relate to the Hu moments of the soil and template contour, respectively. The lower the ratio value is, the closer the contour is to the analysed pattern. Finally, some thresholds are established in Step 24 to discard undesirable soil contours. Given that a set of points defines a contour, the surface contour area was calculated using Green’s theorem. Figure 10d depicts, for instance, contours that surpassed the conditions mentioned above.

A similar contour detection process was applied in Step 27 into the grass layer in order to save those regions and analyse the location relationship between these and the soil contours that were filtered previously. Following Step 31 from Algorithm 1, the distance *d* between grass and soil contour centroids was estimated with Equation (5).

(5) $$d=\sqrt{{\left(P_{x}^{S}-P_{x}^{G}\right)}^{2}+{\left(P_{y}^{S}-P_{y}^{G}\right)}^{2}}$$

where $P_{x}^{S}$ and $P_{y}^{S}$ and $P_{x}^{G}$ and $P_{y}^{G}$ are the spatial *x* and *y* coordinates of the soil and grass contour centroids respectively. Additionally, the minimum intersection linear distances between these contours were calculated to check their proximity, as well. Following Equation (5), the distance between each one of the sets of points that defines those contours was estimated. Ultimately, thresholds were set concerning the calculated variables in Step 34 (Figure 10e), depicting only the ones that satisfied the conditions in the ROI image in RGB colour (Figure 10f).

## 3. Results

In order to test the proposed algorithm, eight cropped hyperspectral images with different characteristics in their scenery were chosen and analysed, the accuracy results of which are shown in Table 1. Those regions include scenes with pre-existing mounds and bush lands in their surroundings, rotated mounds and regions with the absence of mounds. For each one of the cropped images, mounds were visually identified and denoted as samples; the effectively found mounds were named True Positives (TP), undetected mounds as False Negatives (FN) and detected mounds by the algorithm yet missed at the real scene as False Positives (FP). The algorithm’s accuracy was calculated by following Equation (6).

(6) $$Accuracy=\frac{TP}{TP+FN+FP}$$

## 4. Discussion

**Figure 13:**
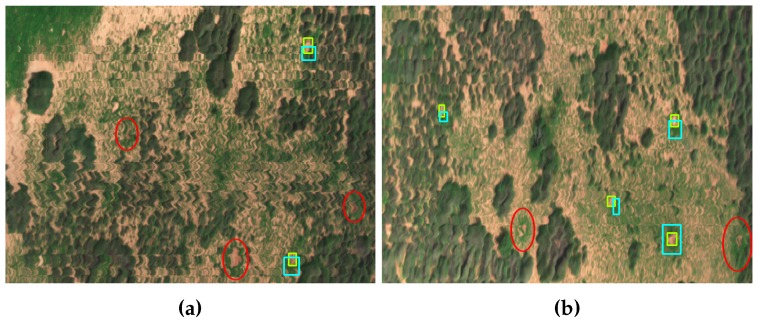
Algorithm outcomes of two ROIs with the remarked missing mounds in red: (**a**) 40% accurate; (**b**) 67% accurate.

By adding an image processing procedure to filter contours by their shapes and identify termite mounds (the segmentation stage of Algorithm 1), the primary outcomes of the proposed method became highly dependent on the GSD values (spatial resolution) and distortion properties from the studied hyperspectral images. As illustrated in Figure 13a, for example, several targets were misclassified owing to a considerable distortion in their oval-based centres and tiny sizes in the scene. Similarly, Figure 13b denotes some limitations in detecting mounds when the oval-based eroded regions are not clearly depicted in the studied area. Both cases revealed how distortion is presented in random areas from any ROI and not only near cornering regions as primarily expected. Conversely, there were successfully identified pre-existing termite mounds regardless of their rotation and location properties in the scene. Additionally, high distortion and low natural light exposure provoked the outcome of false positives, as shown specially for ROI 5 from Table 1. In summary, the given results from detecting appropriately medium and big sized mounds with little distortion presented in the processed images suggest that the proposed algorithm is capable of offering reliable results when decreasing the GSD values and improving the acquisition standards (reducing distortion). However, the unavailability of obtaining new samples at lower altitudes goes beyond the scope of this investigation, and further research is recommended to validate this affirmation.

The Orthorectification Process (ORP) tended to distort some objects’ properties smoothly. Small termite mounds, like the ones depicted in Figure 13, were unlikely to be identified owing to the generated distortion by that process. Incrementing the number of available shape templates might minimise this issue, but conversely, a large number of hyperspectral images would be required to obtain those templates, augmenting, therefore, project costs. As maintained above, acquiring images at lower altitudes might improve the results of the proposed method.

The material clustering process denoted the low viability to identify a unique element signature (spectrum) that could detect termite mounds at the selected sites in Cape Range National Park. Conversely, a minimum of two primary materials was distinguished as shown previously in Figure 9. The main contribution of Algorithm 1 is, therefore, the adequate integration of hyperspectral image processing methods to classify multiple materials and traditional RGB image processing to segment pre-existing termite mounds from that set of material layers. Additionally, the dataset of recovered materials enhances the importance of incorporating hyperspectral technology in similar research.

Amongst the feasible options to optimise the accuracy of Algorithm 1, Evolutionary Algorithms (EA) have been widely used when considering optimisation of multi-objective systems in remote sensing applications [30,31,32]. Through the implementation of EA, input parameters that were manually calculated could now be estimated and optimised. For this case, EA outputs will correspond to the location of the mound regions, and inputs will be composed of all threshold parameters, such as the minimum and maximum contour area, the distance between selected soil and grass contours, the match-shaping tolerance rate and other inner constant parameters from the polygonal approximation method. A set of various algorithms will be treated as population individuals in order to get a resulting one whose threshold values might obtain the best segmentation result.

Working only with RGB digital imagery rather than hyperspectral imaging is challenging or not possible for the task of identifying pre-existing mounds. The available data on digital RGB photos, even though of high resolution, mostly rely on the colour representation of the materials in three bands of the visible spectrum where different vegetation species are symbolised by the same RGB colour representation. The recovered materials spectra of the different vegetation and soil highlight substantial differences on their magnitude from 540–700 nm, 720–800 nm and 924–1000 nm wavelength ranges and small differences at the remaining bands.

## 5. Conclusions

This paper presented an algorithm to detect automatically pre-existing termite mounds by following a pipeline process based on acquired hyperspectral imagery with a UAS, an SVM classifier and shape-based image processing detection methods. Slight differences were found in pixel intensity between many of the green vegetation clustered materials, which meant a challenging task at the classification stage. A combination of “bright grass” and “eroded soil” retrieved material signatures was utilised to classify termite mounds instead of extracting a unique material spectrum for their detection. Results showed an overall accuracy of 68%, in which nearly 27% of the targeted mounds were undetected due to high distortion and low-resolution properties from the hyperspectral dataset. On the other hand, the algorithm misclassified less than 8% of random objects as termite mounds. Due to the execution of shape-based detectors, image resolution is still determinant to obtain accurate results. Nonetheless, judging by regions with similar visible colour representations, hyperspectral imagery demonstrated better capabilities to classify a huge set of materials than implementing traditional segmentation methods on RGB images only. Further research by acquiring samples at lower altitudes, as well as increasing the number of targeted mounds is highly recommended. Additionally, optimisation with EAs will boost the accuracy of the proposed method.

## Figures and Tables

**Table 1:** Accuracy results of the proposed algorithm.

ROI	Termite Mounds	True Positives	False Negatives	False Positives	Accuracy (%)
1	6	5	1	0	83.3
2	9	4	5	1	40.0
3	1	1	0	0	100.0
4	2	1	1	0	50.0
5	13	11	2	3	68.8
6	2	2	0	0	100.0
7	9	7	2	0	77.8
8	2	2	0	0	100.0
9	3	3	0	1	75.0
10	2	2	0	0	100.0
11	4	3	1	0	75.0
12	2	1	1	0	50.0
13	7	4	3	1	50.0
14	1	1	0	0	100.0
15	6	4	2	0	66.7
16	6	5	1	0	83.3
17	7	5	2	0	71.4
18	6	3	3	0	50.0
19	1	1	0	0	100.0
20	7	4	3	1	50.0
21	4	3	1	1	60.0
22	7	5	2	1	62.5
23	3	3	0	0	100.0
24	8	6	2	0	75.0
25	1	1	0	0	100.0
**Total**	119	87	32	9	**68.0%**
**Proportion**	100%	73.1%	26.9%	7.6%	
